# SIRT1/FoxO3 axis alteration leads to aberrant immune responses in bronchial epithelial cells

**DOI:** 10.1111/jcmm.13509

**Published:** 2018-02-07

**Authors:** Serena Di Vincenzo, Irene H. Heijink, Jacobien A. Noordhoek, Chiara Cipollina, Liboria Siena, Andreina Bruno, Maria Ferraro, Dirkje S. Postma, Mark Gjomarkaj, Elisabetta Pace

**Affiliations:** ^1^ Istituto di Biomedicina e Immunologia Molecolare‐Consiglio Nazionale delle Ricerche Palermo Italy; ^2^ Department of Pathology and Medical Biology University Medical Center Groningen University of Groningen Groningen The Netherlands; ^3^ Fondazione Ri.MED Palermo Italy

**Keywords:** bronchial epithelial cells, cigarette smoke, SIRT1/FoxO3, inflammation, immune response, ageing

## Abstract

Inflammation and ageing are intertwined in chronic obstructive pulmonary disease (COPD). The histone deacetylase SIRT1 and the related activation of FoxO3 protect from ageing and regulate inflammation. The role of SIRT1/FoxO3 in COPD is largely unknown. This study evaluated whether cigarette smoke, by modulating the SIRT1/FoxO3 axis, affects airway epithelial pro‐inflammatory responses. Human bronchial epithelial cells (16HBE) and primary bronchial epithelial cells (PBECs) from COPD patients and controls were treated with/without cigarette smoke extract (CSE), Sirtinol or FoxO3 siRNA. SIRT1, FoxO3 and NF‐κB nuclear accumulation, SIRT1 deacetylase activity, IL‐8 and CCL20 expression/release and the release of 12 cytokines, neutrophil and lymphocyte chemotaxis were assessed. In PBECs, the constitutive FoxO3 expression was lower in patients with COPD than in controls. Furthermore, CSE reduced FoxO3 expression only in PBECs from controls. In 16HBE, CSE decreased SIRT1 activity and nuclear expression, enhanced NF‐κB binding to the IL‐8 gene promoter thus increasing IL‐8 expression, decreased CCL20 expression, increased the neutrophil chemotaxis and decreased lymphocyte chemotaxis. Similarly, SIRT1 inhibition reduced FoxO3 expression and increased nuclear NF‐κB. FoxO3 siRNA treatment increased IL‐8 and decreased CCL20 expression in 16HBE. In conclusion, CSE impairs the function of SIRT1/FoxO3 axis in bronchial epithelium, dysregulating NF‐κB activity and inducing pro‐inflammatory responses.

## Introduction

COPD is a chronic inflammatory lung disease with a multifactorial aetiology. Cigarette smoke is the major risk factor for the development of COPD 1, 2.

Inhaled cigarette smoke impairs epithelial barrier function 3, induces damage to proteins and organelles *via* oxidative stress 4 and causes accelerated ageing of the lung 5. As a consequence, the incidence of COPD increases with age and changes in COPD lungs share features with the ageing phenotype. This supports that COPD is a disease characterized by the combination of ‘inflammation and aging’ (named inflammaging) of the lung 5, 6.

Sirtuin‐1 (SIRT1), a NAD^+^‐dependent protein/histone deacetylase, is considered an ‘anti‐ageing’ factor. SIRT1 expression is lower in the lung of patients with COPD compared to healthy controls 7, 8. SIRT1 reduces the process of inflammaging by controlling different mechanisms 9, including deacetylation of forkhead box class O3 (FoxO3) leading to its activation 10. FoxO3 is a transcription factor belonging to the FoxO family, and its expression is lower in the lung of patients with COPD compared to healthy controls 11, 12. Furthermore, SIRT1 and the FoxO proteins interact specifically with the RelA/p65 subunit and inhibit NF‐κB activity 11, 13, 14.

NF‐κB induces the transcriptional activation of several pro‐inflammatory genes involved in immune signalling and inflammatory responses 15. Of these, IL‐8/CXCL8 is a key mediator of neutrophil‐mediated inflammation in the lung of patients with COPD 16. Epithelial cells release IL‐8, especially in response to cell damage 17. CCL20 is another prominent CC chemokine produced by airway epithelium, and its release is altered in COPD 18, 19. CCL20 is specifically chemotactic for Th17 phenotype lymphocytes and displays antimicrobial functions 20, 21.

The role of the SIRT1/FoxO3 axis in the pro‐inflammatory response of bronchial epithelial cells exposed to cigarette smoke, and the consequent changes in the immune responses are largely unknown. Starting from our previous study in which we demonstrated that cigarette smoke decreases nuclear expression and activity of SIRT1 and FoxO3 nuclear expression in bronchial epithelial cells 22, in this study, we want to assess the effects of cigarette smoke exposure on the SIRT1/FoxO3 axis and to unravel the key downstream molecular events that contribute to cigarette smoke‐induced inflammation and the consequent effects on the immune system.

## Materials and methods

### Preparation of cigarette smoke extracts

CSE was prepared as described previously. Briefly, Kentucky 2R4F research‐reference cigarettes (The Tobacco Research Institute, University of Kentucky) were used without filter. Smoke from two cigarettes was bubbled through 25‐ml medium. The extract was prepared freshly, sterilized using a 0.22‐μm filter and used within 30 min. The smoke solution was then adjusted to pH 7.4 and used within 30 min. of preparation. This solution was considered to be 100% CSE and diluted to obtain the desired concentration for each experiment. The concentration of CSE was calculated spectrophotometrically, measuring the OD at the wavelength of 320 nm. The pattern of absorbance, among different batches, showed very little differences, and the mean OD of the different batches was 1.37 ± 0.16. The presence of contaminating LPS on undiluted CSE was assessed by a commercially available kit (Cambrex Corporation, East Rutherford, NJ, USA) and was below the detection limit of 0.1 EU/ml.

### Bronchial epithelial cell cultures

PBECs were obtained by protease digestion from tracheobronchial tissue of five ex‐smoking COPD patients (Table 1) 23 undergoing lung transplantation and from leftover tracheobronchial tissue of six non‐COPD control donor lungs, for whom no further information was available 24, 25. The study protocol was consistent with the Research Code of the University Medical Center Groningen (http://www.rug.nl/umcg/onderzoek/researchcode/index) and national ethical and professional guidelines (htttp://www.federa.org). PBECs were grown in hormonally supplemented bronchial epithelial growth medium (BEGM, Lonza, Walkersville, MD, USA) on collagen/fibronectin‐coated flasks and used for experimentation at passage 2. PBECs were cultured to confluence, hormonally deprived overnight and incubated in the presence and absence of CSE (5%, 10% and 20%) for another 24 hrs, and then, cell lysates were collected for further evaluations.

**Table 1:** Characteristics of patients with chronic obstructive pulmonary disease from whom primary bronchial epithelial cells (PBECs) were obtained

COPD patient	Age (years)	Gender	Smoking status	Packs/year	FEV1 (% predicted)	FEV1/FVC (% predicted)	Stage
1	53	M	ex	40	25	25	GOLD IV
2	60	F	ex	37.5	18	18	GOLD IV
3	54	F	ex	35	25	28	GOLD IV
4	56	M	ex	30	31	29	GOLD III
5	63	F	ex	40	22	32	GOLD IV

M, male; F, female; FEV1, forced expiratory volume in 1 sec.; FVC, forced vital capacity.

The human bronchial epithelial cell line 16HBE was kindly provided by Dr. D.C. Gruenert, University of California, San Francisco. 16HBE cells were maintained in MEM (Gibco, Carlsbad, CA, USA) supplemented with 10% heat‐inactivated foetal bovine serum (FBS; Gibco), 1% MEM non‐essential amino acids (EuroClone), 2 mM L‐glutamine and 0.5% gentamicin (Gibco). Cells were cultured to confluence, then we reduced the serum in the medium from 10% to 1% overnight, and we stimulated the cells with or without CSE (10% and 20%) and Sirtinol (10 μM) for 6 or 24 hrs, and then, respectively, RNA and cell lysates and cell culture supernatants were collected for further evaluations. As internal control, in some experiments, nuclear expression of FoxO3 was assessed with and without overnight serum reduction.

### Western blot

The expression of FoxO3 in 16HBE cells and PBECs and the expression of SIRT1 and NF‐κB in 16HBE cells exposed to the previously described stimuli were evaluated by Western blot analysis. To study SIRT1, FoxO3 and NF‐κB nuclear expression, the protein extracts were treated to separate the cytoplasmic and nuclear protein fractions using a commercial kit ‘NE‐PER Nuclear and Cytoplasmic Extraction Reagents’ following the manufacturer's directions (Thermo Scientific; Waltham, MA, USA). For Western blot analysis, the following antibodies were used: SIRT1 (sc‐15404), FoxO3 (sc‐9812), p65/NF‐κB (sc‐372), Lamin B1 (sc‐377000) from Santa Cruz Biotechnology (Dallas, TX, USA); and β‐actin (A5441—Sigma‐Aldrich, St. Louis, MO, USA). Data are expressed as densitometric arbitrary units by correction with the density of the bands obtained for β‐actin. The quality of nuclear and cytoplasmic protein separation was assessed by measuring Lamin B1.

### Immunoprecipitation and SIRT1 activity

The immunoprecipitation and the measure of SIRT1 activity were performed as previously described 26. SIRT1 was immunoprecipitated from nuclear extracts (100 μg) by overnight incubation with anti‐SIRT1 at 4°C and then incubated with protein A/G agarose beads (sc‐2003—Santa Cruz Biotechnology, Dallas, TX, USA). Then, SIRT1 activity was measured using a commercial kit (BML‐AK500 Enzo LifeSciences, Farmingdale, NY, USA) following the manufacturer's instructions. Deacetylation of the substrate, an acetylated lysine side chain, by SIRT1 and the treatment with the Fluor de Lys^®^ Developer produces a fluorophore. Fluorescence was measured in a microplate reader at a wavelength in the range 350–380 nm and detection of emitted light in the range 440–460 nm.

### Immunoprecipitation of the FoxO3/NF‐κB complex

Anti‐FoxO3 antibody was added to 200 μg of nuclear extracts and incubated overnight at 4°C. Protein A/G agarose beads were added and kept for 2 hrs at 4°C on a rotating rocker. Beads were resuspended in reducing sample buffer for Western blot analysis. The membranes of immunoprecipitated FoxO3 were blotted against the antibodies anti‐p65/NF‐κB and anti‐FoxO3.

### FoxO3 siRNA transfection

16HBE cells were transfected with FoxO3 targeting siRNA or non‐targeting control (On‐Target Plus SmartPool, Dharmacon RNA Technologies, Waltham, MA, USA). Cells were seeded in six‐well plates (1 × 10^5^ cells/well) and incubated at 37°C and 5% CO_2_ in complete growth medium without antibiotics. The transfection was performed according to the manufacturer's instructions using the following conditions: solution A (50 nM siRNA) and solution B (DharmaFECT1, 1:100 dilution). Twenty‐four hours after transfection, medium was removed and MEM with 1% FBS was added for additional 24 hrs and then the samples were collected.

### Real‐time PCR

After 6 hrs of stimulation as above described, total RNA was extracted from 16HBE cells using TRIzol Reagent (Invitrogen, Carlsbad, CA, USA) following the manufacturer's instruction and was reverse‐transcribed into cDNA, using M‐MLV‐RT and oligo(dT)12‐18 primer (Invitrogen). RT‐PCR of IL‐8 and CCL20 transcripts was carried out on Step‐One Plus Real‐time PCR System (Applied Biosystems, Foster City, CA, USA) using specific FAM‐labelled probe and primers (IL‐8 Hs00174103m1 and CCL20 Hs00355476m1, Applied Biosystems). Gene expression was normalized to glyceraldehyde‐3‐phosphate dehydrogenase (GAPDH). Relative quantitation of mRNA was carried out with the comparative *C*_T_ method ($2^{-\Delta \Delta C_{t}}$) and was plotted as relative fold‐change compared to untreated cells that were chosen as the reference sample.

### ChiP assay

ChiP analysis was performed with the EZ‐ChIP kit (Upstate Millipore Corporation, Billerica, MA, USA) following the manufacturer's instructions. The 16HBE cells were stimulated for 24 hrs as above described. The cross‐linked chromatin was sonicated to lengths spanning 200–1000 bp and incubated with the antibody anti‐p65/Nf‐κB [10 μg]. Immunocomplexes were precipitated using protein G agarose. DNA fragments were isolated and purified with columns. PCR was performed with primers specific for IL‐8 gene (forward 5′‐AAGAAAACTTTCTGCATACTCCG‐3′/reverse 5′‐TGGCTTTTTATATCATCACCCTAC‐3′) and CCL20 gene (forward 5′‐TGAGGAAAAAGCAGGAAGTTTT‐3′/reverse 5′‐GTACACAGAAGGCGTGTTGC‐3′).

### Measurement of IL‐8 and CCL20

The concentrations of IL‐8 and CCL20 in cell culture supernatants of 16HBE stimulated for 24 hrs as above described were determined with an enzyme‐linked immunosorbent assays (ELISAs) (DuoSet; R&D Systems, Minneapolis, MN, USA).

### Multi‐analyte ELISArray

For the simultaneous detection of 12 cytokines (IL‐1α, IL‐1β, IL‐2, IL‐4, IL‐6, IL‐8, IL‐10, IL‐12, IL‐17A, IFN‐γ, TNF‐α, GM‐CSF), in cell culture supernatants of 16HBE stimulated for 24 hrs as above described, a Multi‐Analyte ELISArray KIT (Qiagen, Valencia, CA, USA) was used following the manufacturer's instructions.

The expression of each cytokine was expressed as absorbance corrected for dilution of the samples and negative control absorbance was subtracted. Cytokines with a baseline absorbance less than 0.1 were excluded.

### Isolation of peripheral blood mononuclear cells (PBMCs) and neutrophils

PBMCs and neutrophils were isolated from peripheral blood of healthy adult volunteers by one‐step density gradient centrifugation using Polymorphprep (Axis‐Shield) as previously described 27. The different cells were resuspended in RPMI medium without supplement and used immediately after isolation to determinate the actin reorganization.

### Determination of actin reorganization and immunofluorescence

Actin reorganization was assessed by fluorescence microscopy after FITC–Phalloidin (200 ng/ml) staining as previously described 28. Neutrophils and PBMCs (5 × 10^5^/300 μl) were stimulated for 10 min. and 1 hr, respectively, with conditioned medium (diluted 1:10) from 16HBE stimulated with CSE. Cells were fixed (4% paraformaldehyde in PBS), permeabilized (0.1% Triton X‐100 in PBS) and incubated with the fluorescent dye in the dark at room temperature. After 45 min., cells were washed, spotted on slides and analysed by fluorescent microscopy using Axioskop2 Zeiss microscope (Heidelberg, Germany).

### Statistics

Comparison of data from different study groups was performed with the Mann–Whitney test. In the case of comparison of data within a single study group, anova test (Bonferroni test) was used. *P*‐Value <0.05 was accepted as statistically significant.

## Results

### Constitutive FoxO3 expression and its modulation by CSE in PBECs

**Figure 1:**
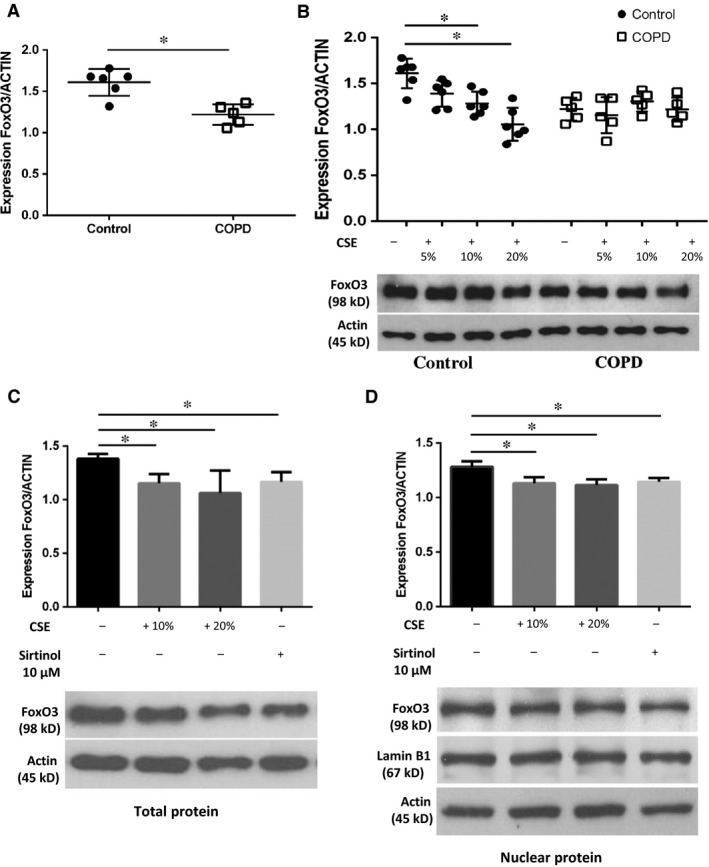
FoxO3 expression in PBECs from COPD patients and healthy controls stimulated with CSE and in 16HBE stimulated with CSE and Sirtinol. PBECs were isolated from COPD (*n* = 5) and from healthy controls (*n* = 6) and assessed for the expression of FoxO3 by Western blot analysis. 16HBE cells were cultured with/without CSE (10% and 20%) and Sirtinol (10 μM) for 24 hrs, and FoxO3 expression was assessed in whole lysates and in nuclear protein extracts. (**A**) Signals corresponding to FoxO3 in unstimulated PBECs were semi‐quantified by densitometric scanning and normalized for β‐actin. (CONTROL: PBECs from healthy individuals; COPD: PBECs from COPD patients). Data were expressed as arbitrary units. Results are expressed mean ± S.D. (**B**) PBECs isolated from COPD (*n* = 5) and from healthy controls (*n* = 6) were cultured with/without 5%, 10%, 20% CSE for 24 hrs. Signals corresponding to FoxO3 were semi‐quantified by densitometric scanning and normalized for β‐actin. (CONTROL: PBECs from healthy individuals; COPD: PBECs from COPD patients). Representative Western blot of FoxO3 and β‐actin (Lanes 1–4: CONTROL stimulated or not with 5%, 10%, 20% CSE; Lanes 5–8: COPD stimulated or not with 5%, 10%, 20% CSE). *P < 0.05 Mann–Whitney test. (**C**) Signals corresponding to FoxO3 from whole lysates were semi‐quantified by densitometric scanning and normalized for β‐actin. Results are expressed as mean ± S.D. (*n* = 7). *P < 0.05 anova (Bonferroni test). Representative Western blot of FoxO3 and β‐actin (lane 1: baseline, lane 2: 10% CSE, lane 3: 20% CSE, lane 4: Sirtinol 10 μM). (**D**) Signals corresponding to the expression of nuclear protein FoxO3 were semi‐quantified by densitometric scanning and normalized for β‐actin. Results are expressed mean ± S.D. (*n* = 6) *P < 0.05 anova (Bonferroni test). Representative Western blot of FoxO3, Lamin B1 and β‐actin (lane 1: baseline, lane 2: 10% CSE, lane 3: 20% CSE, lane 4: Sirtinol 10 μM).

Constitutive protein expression of FoxO3 was significantly lower in PBECs from patients with COPD compared to the levels in cells from healthy controls (Fig. 1A). Furthermore, increasing concentrations of CSE (5%, 10% and 20%) promoted a dose‐dependent reduction in FoxO3 in PBECs from control subjects (Fig. 1B), but not in COPD‐derived PBECs.

### Effects of CSE and Sirtinol on FoxO3 protein expression in 16HBE cells

FoxO3 expression in 16HBE cells (Fig. 1C and D) was dose dependently decreased by 10% and 20% CSE. To evaluate the effect of cigarette smoke and SIRT1 activity on FoxO3 expression, the levels of FoxO3 were evaluated in both whole protein lysates and nuclear protein extracts from 16HBE cells stimulated with CSE (10% and 20%) and in the presence or absence of the SIRT1 inhibitor Sirtinol. CSE and Sirtinol caused a significant reduction in FoxO3 protein in whole lysates and in nuclear extracts (Fig 1C and D). Overnight serum reduction did not affect the nuclear expression of FoxO3 (data not shown).

### Effects of CSE and Sirtinol on deacetylase activity and protein expression of SIRT1 in 16HBE cells

**Figure 2:**
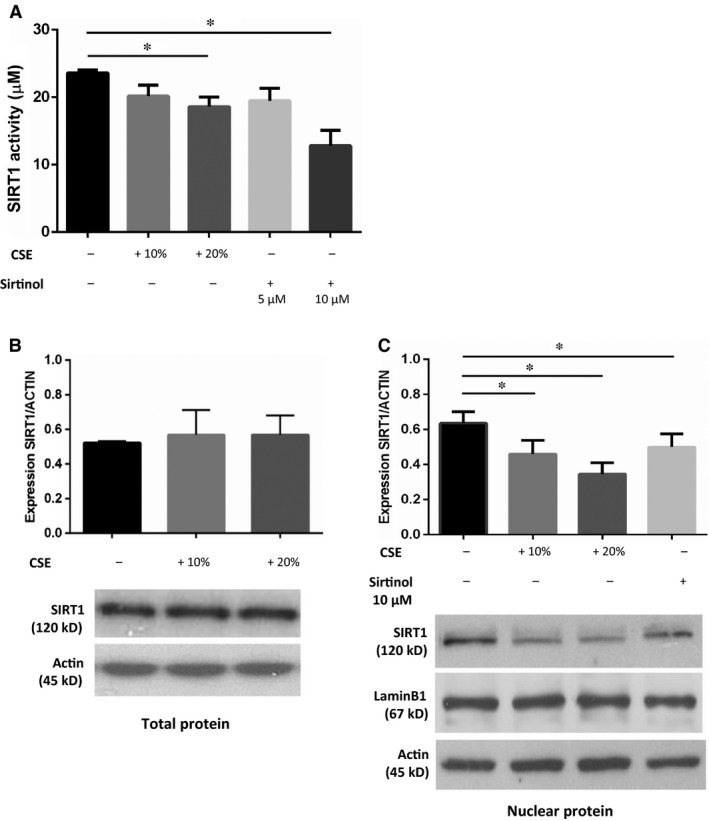
CSE and Sirtinol reduce deacetylase activity and nuclear expression of SIRT1 in 16HBE cells. 16HBE cells were cultured with/without CSE (10% and 20%) and Sirtinol (5–10 μM) for 24 hrs. (**A**) Nuclear proteins were immunoprecipitated and then assessed for SIRT1 activity. Data are expressed as micromolar ± S.D. using as reference a curve of the Fluor de Lys deacetylated standard (provided with the kit). Results are expressed mean ± S.D. (*n* = 5) *P < 0.05 anova (Bonferroni test). SIRT1 protein expression was assessed in whole lysates and in nuclear protein extracts. (**B**) Signals corresponding to SIRT1 from whole lysates were semi‐quantified by densitometric scanning and normalized for β‐actin. Results are expressed mean ± S.D. (*n* = 5) Representative Western blot of SIRT1 and β‐actin (lane 1: baseline, lane 2: 10% CSE, lane 3: 20%). (**C**) Signals corresponding to expression of nuclear protein SIRT1 were semi‐quantified by densitometric scanning and normalized for β‐actin. Results are expressed mean ± S.D. (*n* = 5) *P < 0.05 anova (Bonferroni test). Representative Western blot of SIRT1, Lamin B1 and β‐actin (lane 1: baseline, lane 2: 10% CSE, lane 3: 20% CSE, lane 4: Sirtinol 10 μM).

To further assess the relationship between SIRT1 deacetylase activity and the CSE‐induced reduction in FoxO3 expression, we evaluated the effects of CSE on SIRT1 deacetylase activity and on protein expression in whole protein lysates and in nuclear protein extracts from 16HBE cells. Both 20% CSE and 10 μM Sirtinol significantly reduced SIRT1 activity (20% CSE *versus* baseline: % of reduction of 21.18%, 10 μM Sirtinol *versus* baseline: % of reduction of 45.66%; Fig. 2A). Furthermore, 10% and 20% CSE and 10 μM Sirtinol significantly reduced nuclear SIRT1 expression (10% CSE *versus* baseline: % of reduction of 27.67%, 20% CSE *versus* baseline: % of reduction of 45.60%, 10 μM Sirtinol *versus* baseline: % of reduction of 21.38%; Fig. 2B and C).

### Effects of CSE, Sirtinol and down‐regulation of FoxO3 gene expression on NF‐κB protein expression and effects of CSE on FoxO3 interaction with NF‐κB in 16HBE cells

**Figure 3:**
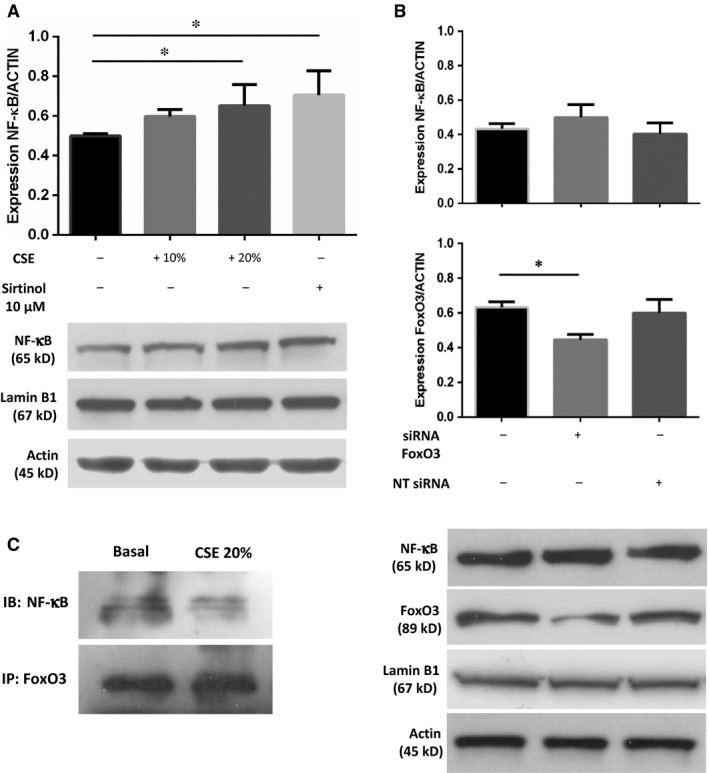
Effects of CSE, Sirtinol and FoxO3 targeting siRNA on NF‐κB expression and effects of CSE on FoxO3/NF‐κB binding in 16HBE cells. (**A**) 16HBE cells were cultured with/without CSE (10% and 20%) and Sirtinol (10 μM) for 24 hrs, and p65/NF‐κB expression was assessed in nuclear protein extracts. Signals corresponding to the expression of nuclear protein p65/NF‐κB were semi‐quantified by densitometric scanning and normalized for β‐actin. Results are expressed as mean ± S.D. (*n* = 5) *P < 0.05 anova (Bonferroni test). Representative Western blot of NF‐κB, Lamin B1 and β‐actin (lane 1: baseline, lane 2: 10% CSE, lane 3: 20% CSE, lane 4: Sirtinol 10 μM). (**B**) 16HBE cells were or not transfected with siRNA FoxO3, and then the nuclear expression of p65/NF‐κB was assessed by Western blot analysis. Signals corresponding to the expression of nuclear protein p65/NF‐κB were semi‐quantified by densitometric scanning and normalized for β‐actin. Results are expressed as mean ± S.D. (*n* = 5) *P < 0.05 anova (Bonferroni test). Representative Western blot of p65/NF‐κB, FoxO3, Lamin B1 and β‐actin. (lane 1: baseline, lane 2: siRNA FoxO3 50 nM + DharmaFECT 1:100, lane 3: non‐targeting (NT) siRNA 50 nM + DharmaFECT 1:100). (**C**) 16HBE cells were cultured with/without CSE 20% for 24 hrs and FoxO3 binding to p65/NF‐κB within the nucleus was assessed by co‐immunoprecipitation of nuclear protein extracts. Representative Western blot of immunoprecipitated FoxO3 (IP) which were blotted against p65/NF‐κB (IB) (lane 1: baseline, lane 2: 20% CSE).

To assess whether cigarette smoke can affect pro‐inflammatory processes by impairing SIRT1/FoxO3 axis, the activation and nuclear translocation of the NF‐κB subunit p65 were evaluated by its expression in nuclear extracts from 16HBE stimulated with CSE (10% and 20%) and Sirtinol. Both CSE 20% and Sirtinol induced nuclear accumulation of p65 (Fig. 3A). The nuclear expression of p65 was not affected by 38% down‐regulation of FoxO3 mRNA using a siRNA approach (Fig. 3B). It has been proposed that FoxO3 regulates the activity of NF‐κB through direct interaction with p65, thus inhibiting its binding to DNA 13. Indeed, co‐immunoprecipitation experiments in 16HBE cells revealed that 20% CSE reduced the interaction between FoxO3 and p65 (Fig. 3C).

### Effects of CSE, Sirtinol and down‐regulation of FoxO3 on IL‐8 and CCL20 gene expression in 16HBE cells

**Figure 4:**
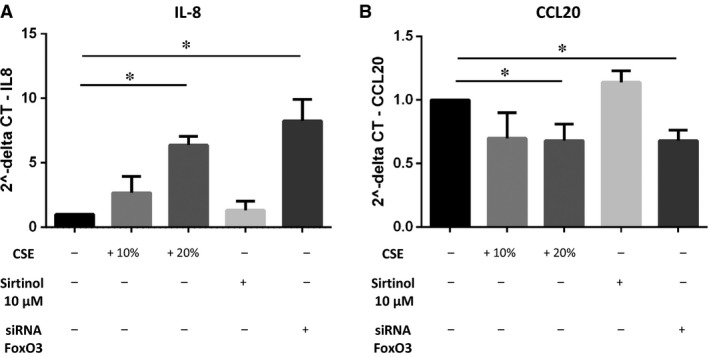
Effects of CSE, Sirtinol and siFoxO3 on the modulation of IL‐8 and CCL20 mRNA in 16HBE cells. 16HBE cells were incubated with increasing concentration of CSE (10% and 20%) and with Sirtinol 10 μM for 6 hrs or 16HBE cells were transfected with FoxO3 targeting siRNA for 48 hrs. Then total RNA was extracted, and real‐time PCR was used to assess IL‐8 (**A**) and CCL20 (**B**) gene expression. GAPDH gene expression was used as endogenous control for normalization. Relative quantitation of mRNA was carried out with comparative CT method. Results are reported as relative unit and normalized to non‐treated control (baseline; *n* = 5). *P < 0.05 anova (Bonferroni test).

Next, we evaluated the consequences of CSE‐induced down‐regulation of SIRT1 and FoxO3 for epithelial pro‐inflammatory activity. Twenty per cent of CSE significantly increased IL‐8 gene expression and reduced CCL20 gene expression in 16HBE cells. In line with a role of FoxO3 in these CSE‐induced effects, cells transfected with FoxO3 targeting siRNA showed the same effects of CSE. Sirtinol did not significantly affect IL‐8 nor CCL20 gene expression (Fig. 4A and B).

### Effects of CSE on NF‐κB binding to promoters of IL‐8 and CCL20 genes in 16HBE cells

**Figure 5:**
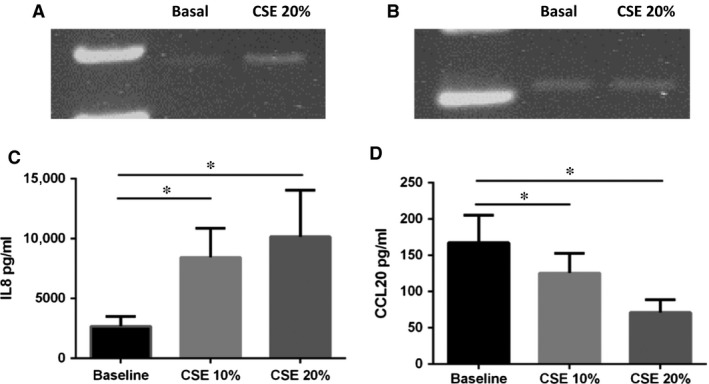
Effects of CSE on NF‐κB binding to IL‐8 promoter and CCL20 promoter gene and on the modulation of IL‐8 and CCL20 release in 16HBE cells. 16HBE cells were incubated with CSE 20% for 24 hrs, and p65/NF‐κB binding to IL‐8 and CCL20 promoters was assessed by ChIP assay. ChIP assay using anti‐p65/NF‐κB antibody and PCR using primers spanning the promoter region of IL‐8 (**A**) and CCL20 (**B**) genes (*n* = 3) (lane 1: baseline; lane 2: CSE 20%). 16HBE cells were incubated with increasing concentration of CSE (10% and 20%), and after 24 hrs, supernatants of stimulated 16HBE cells were collected and the concentration of IL‐8 (**C**) and CCL20 (**D**) was evaluated by ELISA kit. Data are expressed as mean ± S.D. (*n* = 5) *P < 0.05 anova (Bonferroni test).

We further explored whether CSE altered IL‐8 and CCL20 gene expression through the modulation of NF‐κB binding to their gene promoters. As expected, CSE increased the binding of NF‐κB to the promoter of IL‐8 (Fig. 5A) gene, but it did not modify the binding on CCL20 gene promoter (Fig. 5B), suggesting that the CSE‐induced reduction in CCL20 expression is regulated by other nuclear factors.

### IL‐8 and CCL20 concentration in 16HBE supernatants

The effects of CSE on IL‐8 and CCL20 mRNA expression were confirmed at the protein level, showing a significant increase in IL‐8 (Fig. 5C) and decrease in CCL20 (Fig. 5D) protein secretion in 16HBE stimulated with or without CSE (10–20%).

It was also assessed the involvement of other cytokine in this mechanism evaluating the concentration of 12 cytokines (IL‐1α, IL‐1β, IL‐2, IL‐4, IL‐6, IL‐8, IL‐10, IL‐12, IL‐17A, IFN‐γ, TNF‐α and GM‐CSF), in cell culture supernatants of 16HBE stimulated with or without CSE 20% for 24 hrs. All tested cytokines, except IL‐8 and IL‐6, are not detectable in our experimental model. The effect of CSE on the release of IL‐8 was confirmed also with Multi‐Analyte Kit; instead, in this model, CSE has any effect on IL‐6 release (Table 2).

**Table 2:** Cytokines evaluated in cell culture supernatants of 16HBE (*n* = 3)

	IL‐1α	IL‐1β	IL‐2	IL‐4	IL‐6	IL‐8	IL‐10	IL‐12	IL‐17A	IFN‐γ	TNF‐α	GM‐CSF
Baseline	nd	nd	nd	nd	0.175	0.190	nd	nd	nd	nd	nd	nd
CSE 20%	nd	nd	nd	nd	0.165	0.245	nd	nd	nd	nd	nd	nd

IL, interleukin; IFN, interferon; TNF, tumour necrosis factor; GM‐CSF, granulocyte‐macrophage colony‐stimulating factor; nd, not detectable.

### Effects of CSE on actin reorganization in neutrophils and PBMCs

**Figure 6:**
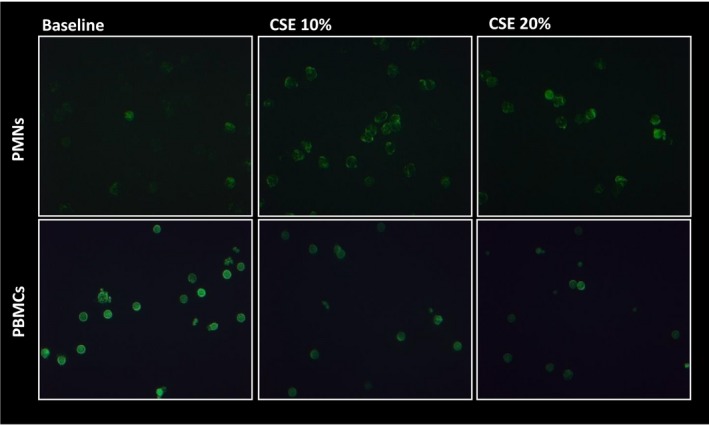
CSE activates actin reorganization in neutrophils (PMNs) and reduces actin reorganization in PBMCs. 16HBE cells were cultured in the presence and in the absence of CSE (10% and 20%) for 24 hrs. The supernatants were collected and used for stimulating neutrophils and PBMCs from normal donors. For assessing actin reorganization in unstimulated and stimulated neutrophils and PBMCs, phalloidin expression was evaluated by fluorescence microscopy.

Finally, the impact of cigarette smoke on modulating the ability of bronchial epithelial cells to secrete neutrophil and lymphocyte chemotactic factors was evaluated. Migration of neutrophils and lymphocytes derives from extracellular‐initiated signalling cascades that orchestrate dynamic reorganization and polymerization of the filamentous actin (F‐actin) in the cytoskeleton 29. The effects of conditioned medium from 16HBE cells treated for 24 hrs with and without CSE (10–20%) on actin reorganization in neutrophils and in PBMCs (predominantly constituted of lymphocytes) were assessed. In response to conditioned medium from 16HBE cells treated with CSE, actin polymerization increased in neutrophils while it decreased in lymphocytes (Fig. 6). Instead, direct stimulation of neutrophils and PBMCs with CSE did not induce any effect (data not shown). These findings suggest that epithelial exposure to CSE promotes chemotaxis of neutrophils.

## Discussion

The airway epithelium is the first barrier towards inhaled insults such as noxious gases and particles present in cigarette smoke and contributes to the inflammation creating a feedback loop that leads to chronic inflammation 28, 30. The present study demonstrates that cigarette smoke exposure reduces FoxO3 expression and that FoxO3 is reduced in airway epithelium of COPD patients. We show that cigarette smoke exposure impairs the function of the anti‐ageing SIRT1/FoxO3 axis, and we demonstrate for the first time that the alteration of SIRT1/FoxO3 axis dysregulates NF‐κB activity, increases IL‐8 and reduces CCL20 expression, thus potentially promoting an imbalance in pro‐inflammatory and antimicrobial responses. This may have important consequences for the development and severity of COPD, as disease exacerbations, sudden worsening of inflammation and symptoms leading to accelerated lung function decline.

The observed constitutive down‐regulation of FoxO3 in COPD‐derived PBECs, compared to PBECs from healthy controls, may be a consequence of chronic cigarette smoking, as confirmed by *in vitro* results. As COPD patients in the current study were ex‐smokers, these effects may be triggered by cigarette smoke and perpetuated over time, probably involving epigenetic changes. FoxO3 down‐regulation may have important implications for COPD, as it regulates the expression of genes involved in several biological processes (oxidative stress, apoptosis, cell cycle regulation, inflammation) 11, 31, 32.

FoxO3 expression and activity are modulated *via* multiple post‐translational modifications. Acetylation of FoxO3 can alter its binding to DNA and transcriptional activity, which can be restored by SIRT1 11, 33, 34. When FoxO3 is not bound to DNA, it can be phosphorylated *via* different cell signalling pathways, leading to the translocation into the cytoplasm and the proteasomal degradation 11, 12, 35. In the current study, 16HBE stimulated with CSE showed reduced levels of FoxO3 in the nucleus and, at the same time, reduced SIRT1 deacetylates activity associated with a reduction in its nuclear protein expression. These results are confirmed also in air–liquid interface culture of 16HBE (data not shown). Furthermore, we found that 16HBE treated with the SIRT1 inhibitor, Sirtinol, showed a decrease in FoxO3 nuclear expression as well as 16HBE treated with CSE. Therefore, we can speculate that the effect of cigarette smoke on FoxO3 expression could be mediated by reduced expression and activity of SIRT1.

Next, we assessed the downstream effects on the inflammatory responses evaluating the involvement of SIRT1 and FoxO3 on NF‐κB mediated pro‐inflammatory responses. The activity of NF‐κB is modulated by multiple mechanisms including control of nuclear accumulation, post‐translational modifications and interactions with co‐regulatory proteins. We showed that CSE induced an increase in NF‐κB nuclear protein expression and that Sirtinol exerted a similar effect. This supported that SIRT1 is involved in the dysregulation of the NF‐κB signalling pathway observed upon CSE exposure. In contrast, silencing of FoxO3 did not reduce NF‐κB nuclear expression in 16HBE cells. We hypothesized that SIRT1 may regulate nuclear accumulation of NF‐κB, while FoxO3 may interact with NF‐κB preventing its binding to DNA. It was demonstrated that the Rel homology domain of NF‐κB interacts with the N‐terminal region of FoxO4 11, 13 that contains the same forkhead DNA‐binding domain that is in FoxO3. Accordingly, we demonstrated for the first time that CSE reduced the interaction between FoxO3 and NF‐κB, thus possibly leading to uncontrolled NF‐κB activation and increased lung inflammatory responses 11, 13, 36.

NF‐κB transcriptionally regulates a large number of genes involved in inflammatory reactions 15 including IL‐8 and CCL20 16, 37. Our results showed that CSE induces an increment of IL‐8 gene expression and release in 16HBE, consistent with the previous literature 17. Regarding CCL20 regulation, contradictory data are available on the effect of cigarette smoke within the airways, probably because CCL20 expression is differently regulated depending on the studied cell types. In total lung homogenate and induced sputum of patients with COPD, CCL20 protein levels were significantly higher compared with never smokers and smokers without COPD 19. On the contrary, studies on CSE‐treated human bronchial epithelial cells 20 and primary human nasal epithelial cells 18 showed reduced CCL20 expression and impaired antimicrobial activity in response to CSE. CCL20 shares its antimicrobial activity with the human β‐defensin (HBD)‐2 which interact with the same membrane receptor, CCR6 38. We have previously shown that CSE reduces HBD2 expression in central airway epithelial cells while increasing it in the epithelium of distal airways of smokers with COPD. The reduction in HBD2 levels is directly correlated with the packs‐year of smoking and is associated with increased airflow obstruction, recurrent infections and increase in exacerbation rates 39. Accordingly, we report that expression and release of CCL20 were down‐regulated in 16HBE stimulated with CSE.

The role of SIRT1/FoxO3 axis in the dysregulation of the IL‐8 and CCL20 expression in bronchial epithelial cells is largely unknown. Our data showed for the first time that FoxO3, but not SIRT1, affects the expression of both chemokines. Furthermore, results from chromatin immunoprecipitation show that CSE induces an increase in the NF‐κB binding to the IL‐8 gene promoter without affecting its binding to the CCL20 promoter. This indicates that CSE induces an imbalance in IL‐8 and CCL20 release not only through the impairment of NF‐κB binding, but also by other mechanisms in which FoxO3, but not SIRT1, is involved. Further experiments will be required to unravel the possible alternative molecular mechanisms.

These differential regulations of IL‐8 and CCL20 prompted us to explore the effects of cigarette smoke‐exposed epithelial cell medium on actin reorganization and thus chemotaxis of neutrophils and lymphocytes, principal players of innate and adaptive immune responses, respectively 40. We found that conditioned medium from CSE‐stimulated 16HBE, but not CSE itself, induced an increase in actin reorganization in neutrophils and a reduction in lymphocytes. This was in line with our previous data showing that CSE increased IL‐8 release and reduced the release of Th1 cell‐attracting chemokine IP‐10 in 16HBE cells, leading to an imbalance between innate and adaptive responses 41. Furthermore, these findings demonstrated that CSE promotes the release of factors that induce a prevalent activation of neutrophils and may thus contribute to neutrophilia of COPD patients.

In conclusion, cigarette smoke impairs the SIRT1/FoxO3 axis, leading to an increase in NF‐κB expression. The imbalance between epithelial production of IL‐8 and CCL20 caused by CSE may result in differential activation of neutrophils and lymphocytes. All these events support a role of cigarette smoke in the balance between pro‐inflammatory/inflammaging response, antimicrobial/innate immune responses and adaptive responses. Such an imbalance may contribute to the chronic inflammation observed in the lung of patients with COPD.

## Authors' Contributions

Serena Di Vincenzo performed the experiments of the study and the statistical analysis of the data, contributed to the writing of the manuscript and declared that she takes the responsibility for the accuracy of the data analysis. Jacobien A. Noordhoek, Chiara Cipollina, Liboria Siena, Andreina Bruno and Maria Ferraro performed the experiments of the study and participated in the interpretation of the data. Irene H. Heijink, Dirkje S. Postma and Mark Gjomarkaj contributed to the interpretation of the data. Elisabetta Pace designed the study, contributed to the interpretation of the data, contributed to the writing of the manuscript and declared that she has had access to and takes responsibility for the integrity of the data. All authors approved the final version of the manuscript.

## Conflict of interest

All authors have no conflict of interest to declare.
